# Squamous Cell Carcinoma Developing in a Cutaneous Lichen Planus Lesion: A Rare Case

**DOI:** 10.1155/2014/205638

**Published:** 2014-03-04

**Authors:** Saptarshi Ghosh, Sivasankar Kotne, P. B. Ananda Rao, S. P. V. Turlapati, Dillip Kumar Soren

**Affiliations:** ^1^Department of Radiotherapy, GSL Medical College & General Hospital, Opposite to Swatantra Hospital, Near Kambala Park, Rajahmundry, Andhra Pradesh 533105, India; ^2^Department of Pathology, GSL Medical College & General Hospital, Rajahmundry, Andhra Pradesh 533296, India; ^3^Department of Surgery, GSL Medical College & General Hospital, Rajahmundry, Andhra Pradesh 533296, India

## Abstract

Lichen planus is a benign disorder characterized by an itchy, noninfectious skin rash. Though lichen planus is a common papulosquamous disorder affecting about 1-2% of the population, neoplastic transformation of cutaneous lichen planus lesions occurs very rarely and should be borne in mind while treating nonhealing longstanding lesions of lichen planus. Studies suggest an estimated 0.3–3% risk of malignancy in patients with oral lichen planus, however, cutaneous lichen planus does not carry an increased risk of malignant degeneration. We present a case of a 36-year-old male with a 10-year-long history of hypertrophic lichen planus who presented with a nonhealing ulcer in the left popliteal fossa. The patient underwent wide local excision with superficial skin grafting. Postoperative histopathological examination revealed verrucous squamous cell carcinoma complicating lichen planus. In view of underlying structure involvement, adjuvant radiation therapy was given. This case is being reported to emphasize the infrequent possibility of development of malignancy in cutaneous lichen planus, especially if it presents as a longstanding, nonhealing, itchy lesion with patchy areas of depigmentation in the lower limbs.

## 1. Introduction

Lichen planus is a benign cell mediated immune response of unknown origin characterized by an itchy, noninfectious skin rash. Though lichen planus is a common papulosquamous benign disorder affecting about 1-2% of the population, neoplastic transformation of cutaneous lichen planus lesions occurs very rarely and should be borne in mind while treating nonhealing longstanding lesions of lichen planus. Studies suggest an estimated 0.3–3% risk of malignancy in patients with oral lichen planus. However, cutaneous lichen planus does not carry an increased risk of malignant degeneration and is not considered as a premalignant lesion unlike its oral counterpart. But few studies have described the occurrence of squamous cell carcinomas from longstanding, nonhealing, itchy lesions of cutaneous lichen planus of the lower limbs [1–5].

## 2. Case Presentation

A 36-year-old male presented with an ulceroproliferative growth in the left popliteal fossa. The lesion in the left popliteal fossa started as a hypertrophic lichen planus lesion which was diagnosed about 10 years back. He had been treated for his lichen planus lesions, but with no signs of relief. There was history of severe itching in the lesion of the left popliteal fossa.

**Figure 1:**
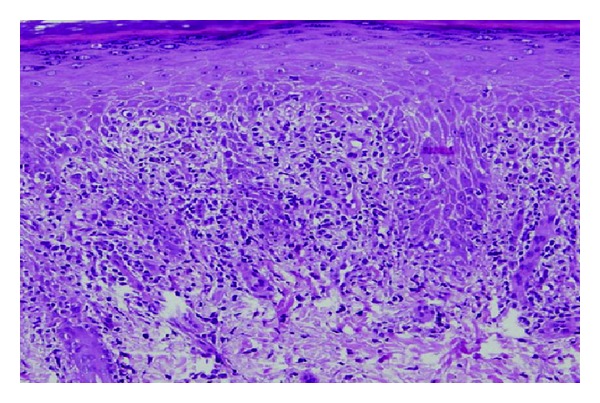
Photomicrograph of hypertrophic lichen planus showing acanthosis, lichenoid mononuclear dermal infiltrate, and colloid bodies (H&E ×40).

A review of the histopathological slide which was made elsewhere 2 years back from the lesion of the left popliteal fossa showed features of hypertrophic lichen planus (Figure 1). On examination, there were multiple pigmented plaques in the dorsum of the foot, shin.

**Figure 2:**
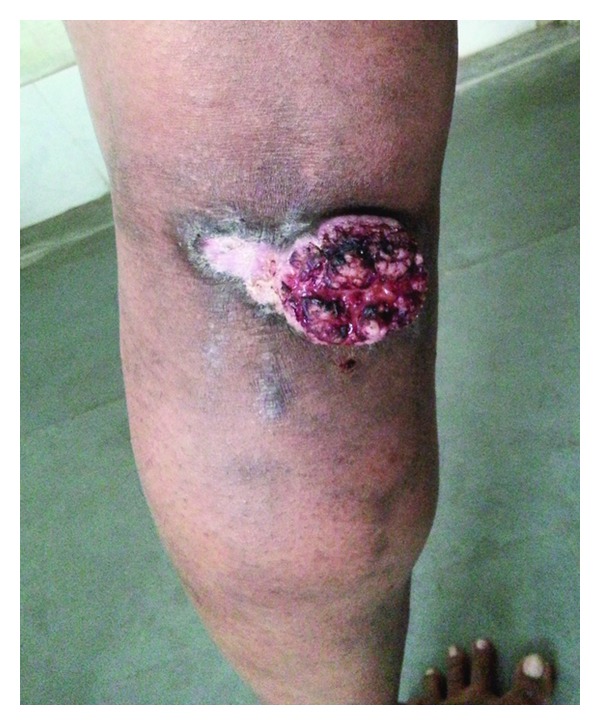
Preoperative picture of the verrucous squamous cell carcinoma developing over a longstanding lesion of hypertrophic lichen planus.

**Figure 3:**
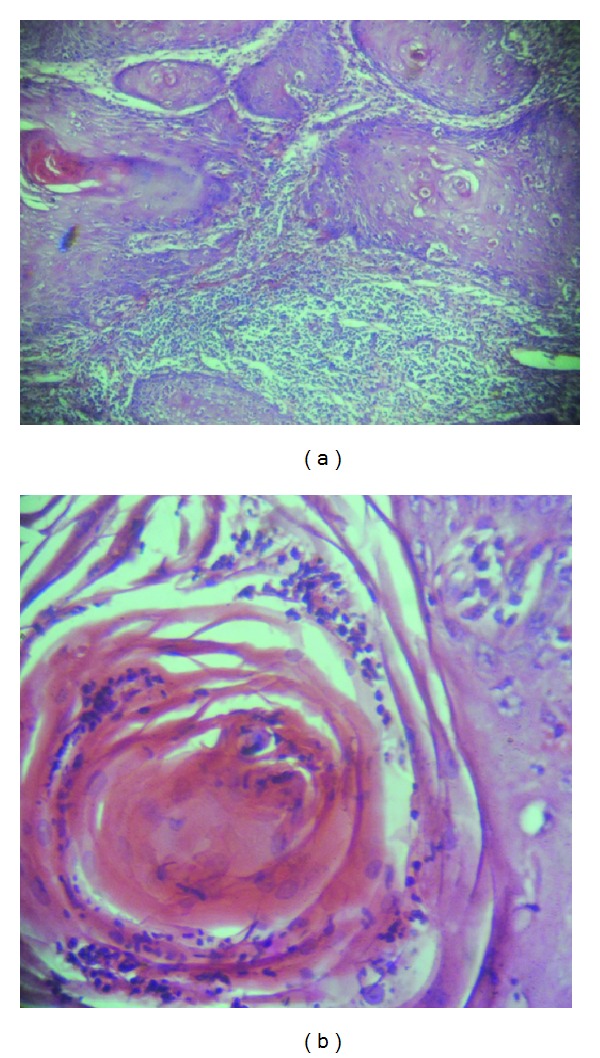
(a) A photomicrograph showing a well-differentiated tumor made up of malignant squamous epithelial cells disposed in islands and cords. Numerous keratin pearls are seen scattered throughout. (b) A photomicrograph showing a keratin pearl in higher magnification. Note: infiltration by neutrophils of the keratin pearl (H&E ×100).

An ulceroproliferative growth of size 7.0 cms × 5.0 cms × 2.5 cms was present on the left popliteal fossa. It was firm in consistency, tender, and with restricted mobility and elevated noneverted margins with areas of depigmentation and hyperpigmentation (Figure 2). He underwent wide local excision with 2 cms margin along with superficial skin grafting from the left thigh. Postoperative histopathological examination revealed a squamous cell carcinoma (Figures 3(a) and 3(b)). In view of underlying structure involvement on biopsy, adjuvant radiation was given to the tumor bed by tangential beams saving the underlying knee joint with the patient lying in prone position. Reexcision was not done as maximum possible resection was already done and the minimal residual disease was close to the major vessels of the lower limbs.

**Figure 4:**
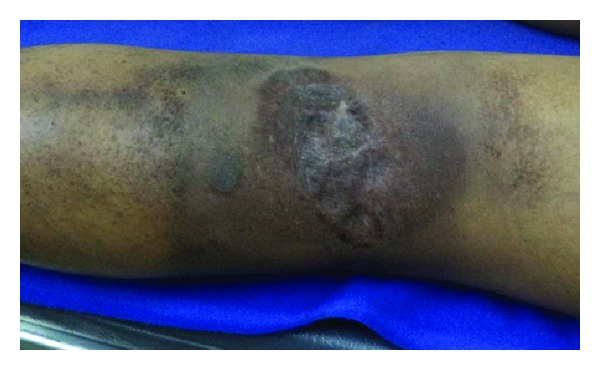
Postirradiation photograph of the left popliteal fossa, there was no joint dysfunction as tangential fields were used in delivering radiation.

The patient is under regular followup thereafter and without any residual disease or local or distant failure at 8 months posttreatment (Figure 4).

## 3. Discussion

Most cutaneous squamous cell carcinomas are associated with risk factors like arsenic exposure, radiation exposure, chronic tar application, ultraviolet rays, burn scars, varicose ulcers, and human papilloma virus [1, 2]. Here in our case the associated risk factors are chronic irritation in the form of itching and longstanding non-healing lesions of lichen planus. There has been a speculation about chronic cutaneous inflammatory lesions triggering an oncogenic-like overdrive of growth factors which stimulate the epithelial cells constantly to undergo neoplastic transformation. While oral lichen planus is considered to be a pre-malignant lesion, no significant association have been yet found between cutaneous lichen planus and squamous cell carcinoma [3]. The incidence of squamous cell carcinomas complicating cutaneous lichen planus is 0.4% and most of the reported cases are of hypertrophic type [4]. Hence it is essential to identify the risk factors of malignant degeneration associated with such cutaneous hypertrophic lichen planus lesions in daily practice so as to diagnose and treat them early in the disease process. Longstanding, nonhealing, severely itchy hypertrophic lichen planus lesions of the lower limbs [1–5] are seen prone to develop malignancy. The presence of areas of depigmentation [1] in the lichen planus lesions has also been noted to be associated with neoplastic transformation, as has been associated in this case too.

This case is being reported to emphasize the role of adjuvant radiation in cases of cutaneous squamous cell carcinomas developing from longstanding, nonhealing, itchy cutaneous lichen planus lesion in the lower limbs. Therefore, such lesions should be subjected to regular histopathological examinations for early detection of skin cancers. Adjuvant radiation is indicated when reexcision of the tumor is not possible, when the residual disease is very close to major neurovascular structures. Adjuvant radiation therapy either with electrons or photons has been shown to reduce the local and distant failure rates in such cases. Posttreatment followup is essential to detect any recurrences or even a second squamous cell carcinoma arising from a different lichen planus lesion.
